# Electromembrane Extraction of Posaconazole for Matrix-Assisted Laser Desorption/Ionization Mass Spectrometric Detection

**DOI:** 10.3390/membranes12060620

**Published:** 2022-06-14

**Authors:** Chi-Sheng Chen, Wen-Chi Chen, Sarah Y. Chang

**Affiliations:** 1 Department of Chemistry, National Kaohsiung Normal University, No. 62, Shenjhong Rd., Yanchao, Kaohsiung 824, Taiwan; light75442@gmail.com; 2 Division of Gastroenterology and Hepatology, Department of Medicine, Kaohsiung Veterans General Hospital, 386 Ta-Chung 1st Road, Kaohsiung 813, Taiwan; wcchen@vghks.gov.tw; 3 Faculty of Medicine, School of Medicine, National Yang Ming Chiao Tung University, Taipei 112, Taiwan; 4 Institute of Biomedical Sciences, College of Science, National Sun Yat-sen University, Kaohsiung 804, Taiwan

**Keywords:** electromembrane extraction, matrix-assisted laser desorption/ionization mass spectrometry, basic drug, posaconazole

## Abstract

A new mode of electromembrane extraction (EME) has been developed for detection via matrix-assisted laser desorption/ionization mass spectrometry (MALDI/MS). Posaconazole, extracted from 8 mL of a 10 mM trifluoroacetic acid solution onto a thin polyvinylidene difluoride (PVDF) membrane, was used as a model analyte. The transport was forced by an electrical potential difference between two electrodes inside the lumen of a hollow fiber and glass tube. Under an application of 80 V, cationic posaconazole in the sample solution moved toward the negative electrode inside the glass tube and was trapped by the PVDF membrane on the side. After 15 min of extraction, 3 μL of α-cyano-4-hydroxycinnamic acid (CHCA) solution was applied on top of the membrane, which was then analyzed by MALDI/MS. Under optimal extraction conditions, the calibration curve of posaconazole was linear over a concentration range of 0.10–100.00 nM. The limit of detection (LOD) at a signal-to-noise ratio of 3 was 0.03 nM with an enhancement factor of 138 for posaconazole. The application of this method to the determination of posaconazole in human serum samples was also successfully demonstrated.

## 1. Introduction

Trace analysis of target analytes in complicated sample matrices is a challenge in chemical analysis. In the past two decades, substantial efforts have been made toward attaining faster, simpler, less expensive, and more environmentally friendly sample preparation methods [1]. Electromembrane extraction (EME), first described by Pedersen-Bjergaard et al. in 2006 [2], is one of the most recently reported techniques that meets these requirements. EME has become a popular technique because of its rapidity, low cost, ease of operation, high selectivity, and low consumption of organic solvents.

The acceptor solution of EME is aqueous; thus, it is compatible with high-performance liquid chromatography (HPLC) and capillary electrophoresis (CE). EME is most commonly coupled to LC, with either UV [3,4] or MS detection [5,6]. Because both the donor and acceptor solutions in EME are aqueous, EME is suitable for coupling to LC, especially reversed-phase LC. Usually, the acceptor solution is collected after the extraction, transferred to a sample vial, and injected into LC. EME is also suitable for coupling to CE due to the compatibility of the acceptor solution with the CE system [7,8], which is more suitable than reversed-phase LC for separating hydrophilic and charged analytes. EME coupled with LC and CE were applied to the analysis of drug compounds [4,5,7,8], amino acids [9], herbicides [10], and peptides [11,12]. It is challenging to combine EME with gas chromatography (GC) due to difficulties arising from the direct injection of the aqueous acceptor phase. To combine it with GC, a two-phase EME using an organic acceptor phase was reported [13]. To use an aqueous acceptor solution, solid-phase microextraction (SPME) [14], dispersive liquid–liquid microextraction (DLLME) [15], and liquid–liquid microextraction (LLME) [16] must be performed after EME extraction. EME coupled to GC has been applied to the analysis of drug compounds [16], phthalate compounds [13], and biogenic amines [17]. EME followed by voltammetry to extract basic [18] and acidic drugs [19] has also been reported. Additionally, heavy metal ions were successfully extracted by EME and analyzed by atomic absorption spectrometric [20,21] and fluorescence detection [22]. Previously, solvent-free EME coupled to matrix-assisted laser desorption/ionization mass spectrometry (MALDI/MS) to determine peptides was developed in our laboratory [23]. However, the system was operated with a high current of up to 5 mA, which is substantially higher than typically recommended (1–400 μA) [24]. For biological fluid samples, the system current was too high and resulted in the breakdown of the SF–EME system due to the high salt content in the samples. Therefore, solvent-free EME cannot be applied to samples with complicated matrices.

Posaconazole is a second-generation triazole fungicide that has become the most widely prescribed medication to treat invasive fungal infections [25,26]. A threshold of >0.5–0.7 μg/mL has been recommended as concentration in plasma to ensure treatment efficacy [27]. MALDI/MS has been extensively employed to characterize low molecular weight compounds. However, samples containing complicated matrices suffer from ionization suppression. This study developed a new mode of electromembrane extraction (EME) for detection via MALDI/MS, and for this the basic drug posaconazole was used as a model analyte. The parameters affecting extraction efficiency and detection were optimized, and the method’s applicability for detecting posaconazole in human serum was demonstrated. To the best of our knowledge, this is the first report that demonstrates the use of EME coupled with MALDI/MS to determine a basic drug.

## 2. Materials and Methods

### 2.1. Chemicals and Solutions

Posaconazole, 2-nitrophenyl octyl ether (NPOE), and α-cyano-4-hydroxycinnamic acid (CHCA) were purchased from Sigma-Aldrich (St. Louis, MO, USA). Trichloroacetic acid (TFA) was obtained from Alfa-Aesar (Ward Hill, MA, USA). Hydrophilic polyvinylidene difluoride (PVDF), nylon, polytetrafluoroethylene (PTFE), mixed cellulose ester (MCE), and hydrophobic polypropylene (PP) sheet membranes with a thickness of 200 μm and a pore size of 0.22 μm were purchased from Greattech Technology Ltd. (Taipei, Taiwan). All chemicals were used as received without further purification. Water was purified with a Millipore Synergy water purification system (Billerica, MA, USA) and used for all experiments.

A stock standard solution (1 mM) of posaconazole was prepared in methanol and diluted to the desired concentrations with a 10 mM TFA aqueous solution (pH 2.2). The posaconazole solutions were stored at −20 °C and protected from light for three months. CHCA solution (10 mg/mL) was freshly prepared in a 50% methanol/water solution containing 0.1% TFA.

### 2.2. EME Procedure

**Figure 1:**
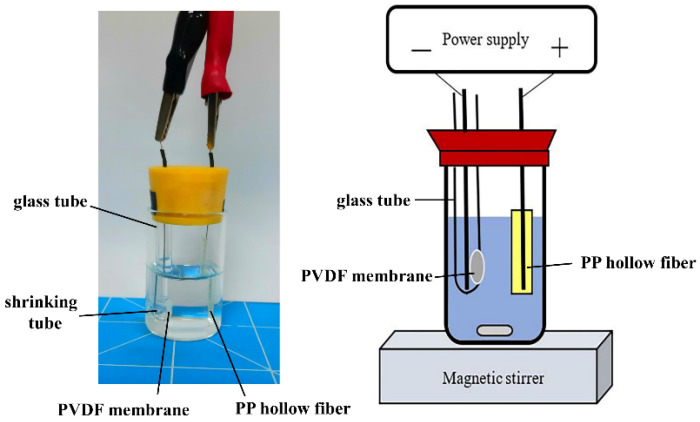
Schematic illustration of the EME setup.

A schematic diagram of the EME setup is illustrated in Figure 1. A glass vial (15 mL) was employed as the sample compartment. A glass tube with an internal diameter of 3.3 mm (48 mm height) was fixed to the left side of a rubber cap. A 4.7 mm diameter PVDF membrane with a thickness of 200 μm and a pore size of 0.22 μm was cut and fixed to the side of the glass tube by a heat shrinking tube. A 3.0 cm piece of PP hollow fiber with a thickness of 200 μm and a pore size of 0.22 μm (diameter 3.0 mm) was dipped in NPOE for 10 s and served as the SLM. Subsequently, 50 μL of 10 mM TFA was injected into the lumen of the hollow fiber with a microsyringe. The hollow fiber containing SLM was inserted through a hole on the right side of the rubber cap of the sample compartment. Two platinum wires (diameter 0.5 mm and length 6.8 cm) were used as electrodes with an interelectrode distance of 1.5 cm. The cathode and anode were placed into the lumen of the hollow fiber and glass tube, respectively, and connected to the DC power supply (P-200, Hila International Inc., Hsinchu, Taiwan) with a variable voltage in the range of 0–200 V. The EME system current was converted to voltage by a 1 kΩ resistor. Current measurements were recorded with a computer connected to the Peak-ABC chromatographic data handling system (Great Tide Instrument Company, Taipei, Taiwan).

An 8 mL aliquot of posaconazole standard solution was placed into the sample compartment. When the glass tube was put into the solution, the posaconazole flowed through the hydrophilic PVDF membrane into the tube. The solution was stirred at an agitation rate of 100 rpm throughout the experiments, which were performed at room temperature. Extraction was initiated by the application of 80 V. Under the applied voltage, cationic posaconazole in the sample solution moved toward the negative electrode and was trapped by the membrane on the side of the glass tube. After extraction, the shrinking tube was removed, and the posaconazole-trapped membrane was transferred onto the MALDI sample target, front side up.

### 2.3. MALDI/MS Measurements

The overlayer technique was used to prepare the MALDI sample. The posaconazole-trapped membrane was deposited onto a stainless steel target using double-sided tape and air-dried at room temperature. Then, 3 μL of CHCA solution was applied to the top of the membrane prior to MALDI/MS analysis. For regular MALDI samples, 1 μL of posaconazole solution mixed with 1 μL of CHCA solution was deposited onto a stainless steel target and allowed to dry at room temperature.

Mass spectrometry experiments were performed in positive-ion mode on a reflectron-type time-of-flight mass spectrometer (Microflex, Bruker Daltonics, Bremen, Germany) with a flight length of 1.96 m. The samples were irradiated with a 337 nm nitrogen laser at 30 Hz. The generated ions were accelerated at a voltage of 19 kV. To obtain good signal-to-noise ratios, the laser energy settings were adjusted to slightly exceed the threshold, and each spectrum was acquired from an average of 200 laser pulses.

### 2.4. Preparation of Human Serum Sample

Drug-free human serum was purchased from Sigma-Aldrich (St. Louis, MO, USA) and stored in plastic tubes at –20 °C. An aliquot (990 μL) of the serum sample was spiked with 10 μL of the posaconazole standard. Serum samples with various posaconazole concentrations were prepared by spiking the serum with the desired amounts of posaconazole. A blank serum sample was prepared by spiking 990 μL of serum sample with 10 μL of DI water. The serum sample pretreatment procedure was a modification of a previously reported method [28]. A 1 mL aliquot of posaconazole-spiked serum sample was maintained in a 70 °C oven for 10 min. The serum sample was then deproteinized by adding 1 mL of 130 mM TFA solution. After shaking, the serum sample was again maintained in a 70 °C oven for 10 min. After centrifuging at 6000× *g* for 10 min, 1 mL of the supernatant liquid was diluted to a total volume of 8 mL with 10 mM TFA solution. Serum samples were then subjected to EME according to the procedure described above.

## 3. Results and Discussions

### 3.1. Detection of Posaconazole on Membrane by MALDI/MS

**Figure 2:**
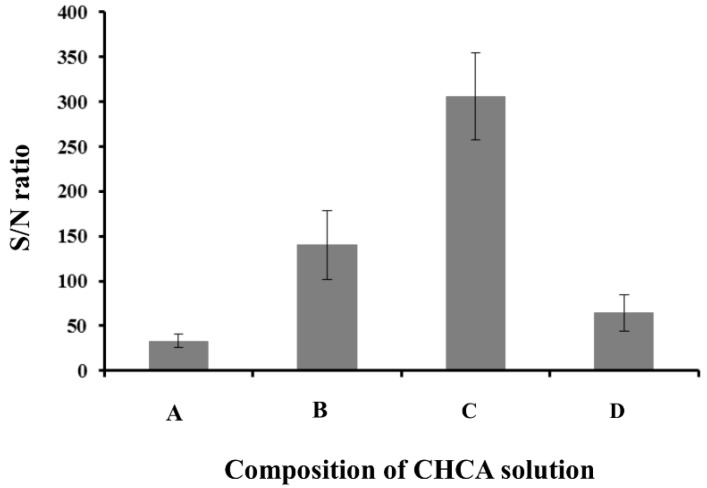
Effect of CHCA solution composition on the S/N ratio of posaconazole. CHCA solution in 0.1% aqueous TFA contained (**A**) 30% acetonitrile, (**B**) 30% methanol, (**C**) 50% methanol, and (**D**) 70% methanol. Experimental conditions: sample volume, 3 μL; CHCA solution volume, 3 μL; and posaconazole concentration, 1.00 μM. A total of 200 pulsed laser shots were applied using a laser adjusted to energy levels that slightly exceeded the threshold.

The membrane’s primary function is to trap analytes from the sample solution. In addition, the analyte–MALDI matrix mixture should form homogeneous crystals on the membrane. Therefore, membrane selection is critical for successful MALDI/MS detection. Different types of commercially available membranes were evaluated. Hydrophilic nylon, PTFE, MCE, and PVDF membranes were tested. To test if posaconazole can be desorbed and ionized from the commercially available membranes, 3 μL of posaconazole solution was deposited onto the membrane and allowed to dry at room temperature. Then, 3 μL of CHCA solution in 30% acetonitrile/water solution containing 0.1% TFA was applied to the membrane. No posaconazole ions were observed with nylon, PTFE, or MCE membranes; however, on the PVDF membrane it was successfully desorbed and ionized with an ion signal at *m*/*z* = 701.78, which corresponds to the protonated molecular ion of posaconazole (Figure 2A). However, the spot-to-spot reproducibility of the sample preparation varied by 24.0% over 21 sample spots (3 samples). When applying CHCA solution to the membrane, posaconazole was redissolved in CHCA solution and underwent homogeneous crystallization with CHCA on the PVDF membrane. The homogeneity of the analyte’s crystallization and signal-to-noise ratio (S/N ratio) were also affected by the CHCA solution’s composition. Therefore, choosing an appropriate organic solvent in the solution is important for obtaining a satisfactory MALDI/MS detection for posaconazole. Since it is soluble in methanol, a CHCA solution in a 30% methanol/water solution containing 0.1 TFA was tested. As shown in Figure 2B, the S/N ratio of posaconazole was 4.5 times higher than for a S/N ratio using a CHCA solution in 30% acetonitrile/water solution. The effect of methanol content in the CHCA solution was also investigated from 30% to 70%. As shown in Figure 2, the results indicated that CHCA in a 50% methanol/water solution provided the highest S/N ratio of posaconazole. The spot-to-spot reproducibility of the sample preparation varied by less than 6.0% over 21 sample spots (3 samples). Therefore, CHCA in a 50% methanol/water solution was selected for subsequent experiments.

### 3.2. Optimization of EME Conditions

The pH in the sample solution is critical. To maintain the protonation of an analyte, the general recommendation is to adjust the pH in the sample solution to below the analyte pKa value. According to the literature, the pKa values of posaconazole·H^+^ are 3.6 and 4.6 [29,30]; therefore, trifluoroacetic acid (10 mM, pH 2.2) was used as the background electrolyte in the sample solution. At pH 2.2, posaconazole carries two positive charges; therefore, the negative electrode was placed inside the glass tube, and the positive electrode was placed inside the lumen of the hollow fiber. Upon applying an electrical potential, positively charged posaconazole migrated toward the negative electrode inside the glass tube and was trapped by the membrane on the side of the glass tube under the action of an electric field.

**Figure 3:**
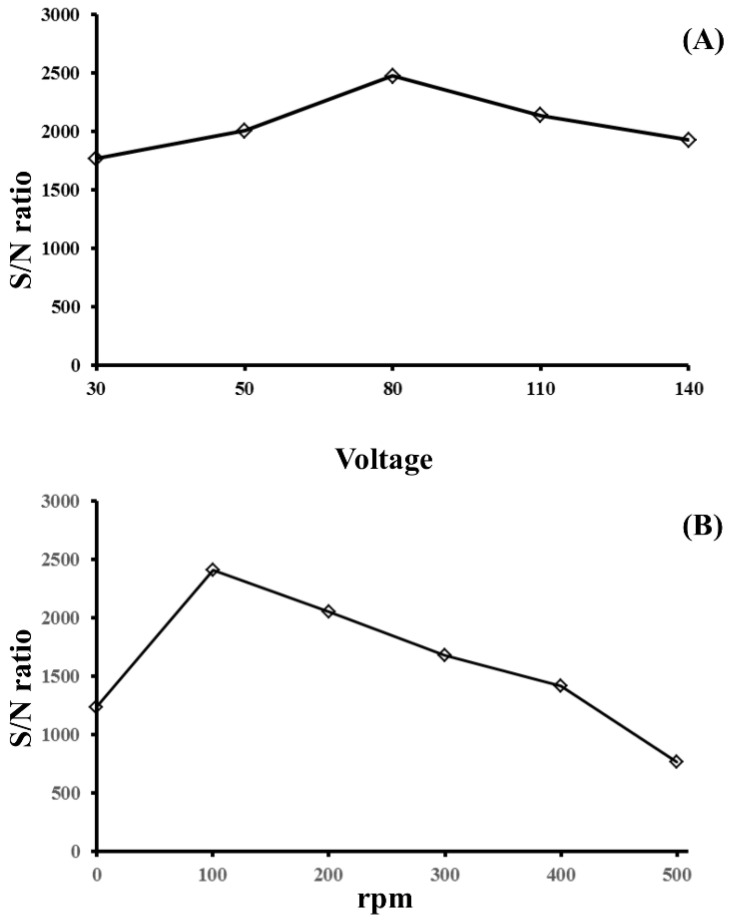
Effect of (**A**) the applied voltage and (**B**) the stirring rate on the S/N ratio of posaconazole obtained from EME. Experimental conditions: sample volume, 8 mL; extraction time, 5 min; and posaconazole concentration, 0.50 μM. All other conditions were identical to those outlined in Figure 2.

Different experimental factors were studied to optimize the efficiency of the extraction procedure. Each data point was calculated by averaging the S/N ratio of 21 sample spots (three samples). The applied voltage was investigated in the range of 30 to 140 V at an extraction time of 5 min. As shown in Figure 3A, the posaconazole S/N ratio increased when the applied voltage increased from 30 to 80 V. A further increase from 80 to 140 V showed a decrease in the S/N ratio. When the voltage was increased, the system current increased, which led to unwanted electrolysis at the electrode and caused the extraction efficiency to decrease. Thus, an applied voltage of 80 V was used in the following experiments.

**Figure 4:**
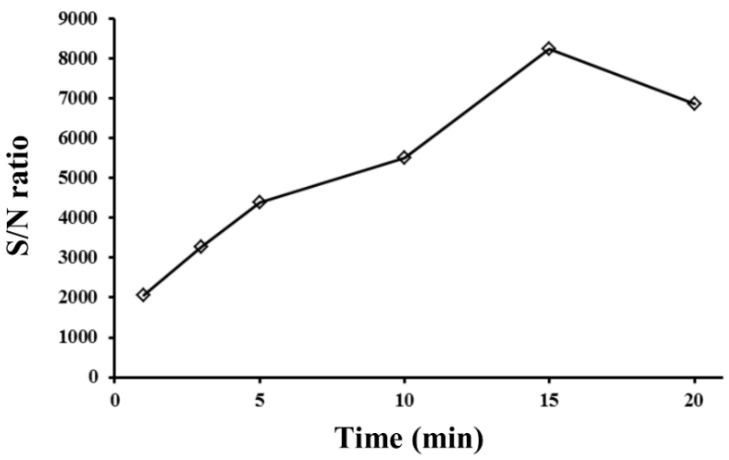
Effect of extraction time on the S/N ratio of posaconazole obtained from EME. Experimental conditions: sample volume, 8 mL; applied voltage, 80 V; stirring rate, 100 rpm; and posaconazole concentration, 0.50 μM. All other conditions were identical to those outlined in Figure 2.

The mass transfer of the charged posaconazole onto the PVDF membrane was driven by diffusion and electromigration [31]. Stirring also enhanced mass transfer during extraction. To optimize the extraction efficiency, the agitation of the sample solution was carried out with varying stirring rates ranging from 0 to 500 rpm. The effect of sample agitation rate on the S/N ratio is shown in Figure 3B. The S/N ratio of posaconazole increased when the stirring rate increased from 0 to 100 rpm and then decreased with increasing agitation rate. The S/N ratio obtained at a stirring speed of 100 rpm was increased only two-fold compared to no stirring. The results showed that electrokinetic migration is the main driving force for the mass transfer of charged posaconazole onto the PVDF membrane. Therefore, 100 rpm was selected as the best sample agitation rate for extracting posaconazole. The effect of extraction time on the S/N ratio of posaconazole was investigated by varying the extraction time from 1 to 20 min. The S/N ratio of the analyte increased when the extraction time increased from 1 to 15 min. A further increase in extraction time decreased the S/N ratio, as shown in Figure 4. The analyte’s penetration might cause a decrease in the S/N ratio of posaconazole through the PVDF membrane’s pores into the solution of the glass tube. Therefore, the amount of analyte on the PVDF membrane decreased. The extraction time was set to 15 min for subsequent experiments.

### 3.3. Analytical Characteristics

**Figure 5:**
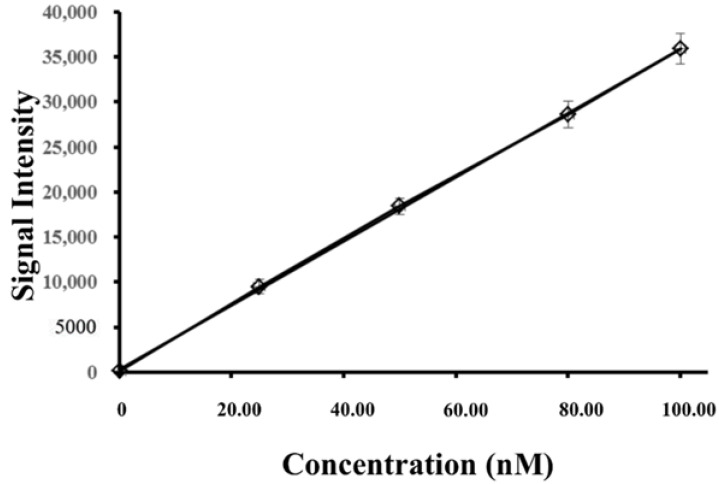
Calibration curve of posaconazole in an aqueous solution under optimized conditions.

**Figure 6:**
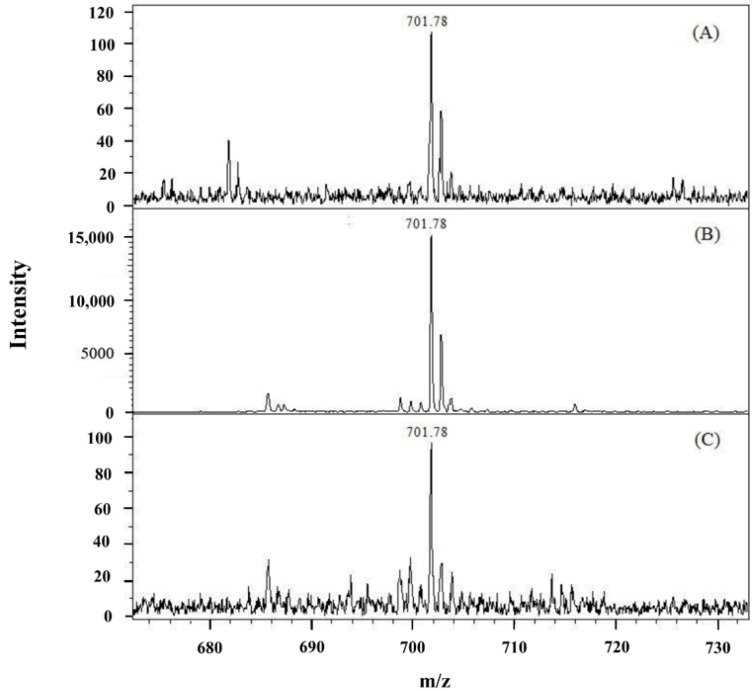
Mass spectra of (**A**) posaconazole (25.00 nM) obtained without EME, (**B**) posaconazole (25.00 nM) obtained with EME, and (**C**) posaconazole (0.10 nM) obtained with EME. The ion signal at *m*/*z* = 701.78 corresponds to the protonated molecular ion of posaconazole. Experimental conditions: sample volume, 8 mL; applied voltage, 80 V; stirring rate, 100 rpm; and extraction time, 15 min. All other conditions were identical to those outlined in Figure 2.

A calibration curve for posaconazole in an aqueous solution was constructed over the concentration range of 0.10–100.00 nM, as shown in Figure 5. A plot of the posaconazole signal intensity versus concentration presented good linearity (*y* = 376.2*x* + 14.3) with a correlation coefficient (r) of 0.9989. The limit of detection (LOD) was calculated based on a signal-to-noise (S/N) ratio of 3 and was 0.03 nM for posaconazole. Figure 6A shows the mass spectrum of posaconazole (25.00 nM) without extraction. The S/N ratio of posaconazole was determined to be 14. With the use of EME to preconcentrate posaconazole, the signal intensities were greatly enhanced, with an S/N ratio of 1938, as shown in Figure 6B. The enhancement factor (EF) of EME was calculated according to previously reported methods [32]. The EF of posaconazole was calculated to be 138. The mass spectrum of the lowest concentrations of posaconazole (0.10 nM) with EME is shown in Figure 6C.

### 3.4. Analysis of Serum Samples

**Figure 7:**
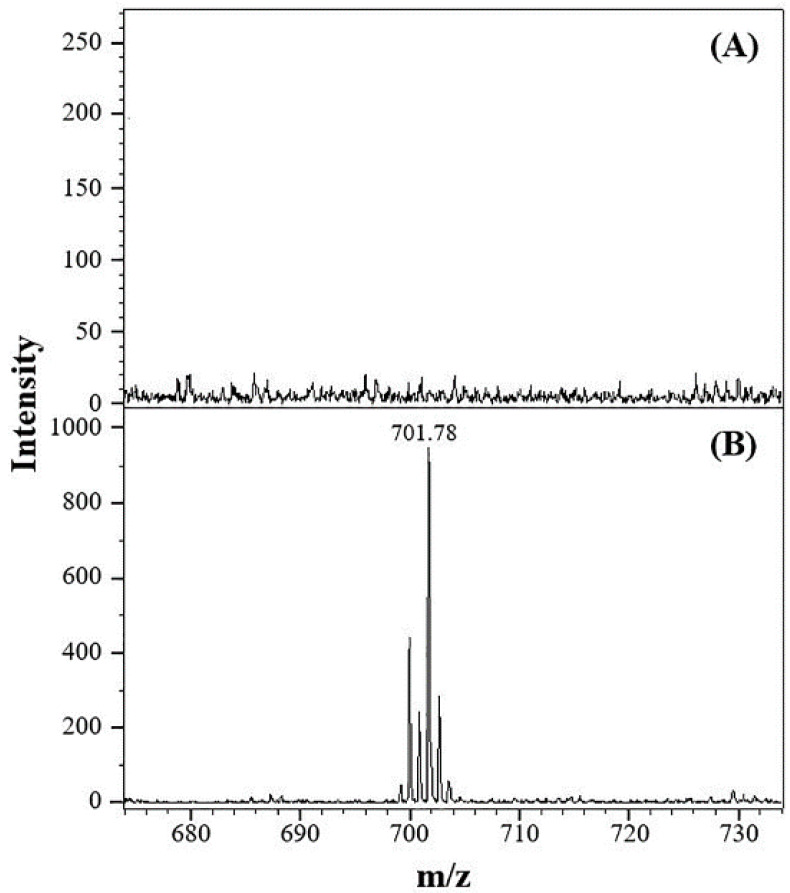
Mass spectra of human serum samples. (**A**) Posaconazole-spiked serum without EME and (**B**) posaconazole-spiked serum with EME. The ion signal at *m/z* = 701.78 corresponds to the protonated molecular ion of posaconazole. Experimental conditions: sample volume, 8 mL; applied voltage, 80 V; stirring rate, 100 rpm; extraction time, 15 min; and posaconazole, 0.80 μM. All other conditions were identical to those outlined in Figure 2.

To evaluate the applicability of this method for biological and clinical analyses, human serum spiked with posaconazole was used as test samples. Because of the high protein content, deproteinization of the serum samples prior to EME was required. Then, one milliliter of the serum sample was diluted to 8 mL with the 10 mM TFA solution and treated using the EME technique with the above procedure. A direct analysis (without EME) of the posaconazole-spiked serum sample was performed. No posaconazole ions were detected without EME, as shown in Figure 7A. Samples from biological sources often contain high salt concentrations, and the presence of these salts significantly suppresses the analyte signals in MALDI/MS. When using EME to extract posaconazole from the posaconazole-spiked serum sample, posaconazole was detected in the mass spectrum (Figure 7B). The signal at *m*/*z* = 701.78 corresponded to the protonated ions of posaconazole. The calibration curve showed good linearity over the concentration range of 0.24–16.00 μM with a correlation coefficient (r) of 0.9986 for posaconazole in serum. The LOD for posaconazole in serum was calculated to be 0.07 μM. The linear range of posaconazole sufficiently covered the therapeutic concentration range encountered in serum (0.71 to 1.70 μM) [33].

The intraday and interday accuracy and precision of the method were evaluated using serum samples spiked with low (0.5 μM), medium (5.0 μM), and high (15.0 μM) concentrations of posaconazole. The results are presented in Table 1. The intraday and interday accuracies for the three spiked levels of posaconazole in serum were −4.0–9.1% and 1.4–8.9%, respectively. The intraday and interday precisions for the three spiked levels of the two analytes were 9.3–11.3 and 7.4–12.5%, respectively. The recovery of posaconazole from serum samples was determined by a standard addition method. Based on triplicate measurements, the mean recovery of posaconazole in serum samples was 8.4% because it is a nonpolar molecule, exhibiting 90–99% serum protein-binding in human serum [34], leading to low recovery in serum samples.

Compared with previous methods for posaconazole quantification in human plasma or serum samples, the LOD of posaconazole obtained here was lower. In particular, our result was lower than the voltammetric method using a carbon nanotube-screen printed electrode [35] and comparable to those reported using CE coupled to SPE, sample stacking techniques [36,37], and LC coupled to fluorescence detection [38]. Although LC/MS and LC/MS/MS provided relatively low LODs for posaconazole, elaborate sample preparation procedures and sophisticated instrumentation were required [39,40]. Moreover, a mass spectrum can be obtained within 10 s using MALDI/MS. Whereas a mass spectrum using LC/MS might need at least 10 min. An important advantage of this newly developed technique is its simplicity and analytical speed. The EME of posaconazole can be performed in 15 min. The data acquired and shown in each mass spectra are the average of 200 spectra, which are the result of 200 laser shots acquired in 10 s. The analytical speed, ease of operation, and high sensitivity allow MALDI/MS with EME to be used for clinical monitoring of the posaconazole content in human serum samples.

## 4. Conclusions

In this work, a new EME mode was developed to extract a basic drug followed by MALDI/MS detection. For the first time, EME in combination with MALDI/MS was used for the determination. After a 15 min extraction, the PVDF membrane was directly analyzed by MALDI/MS, and the mass spectrum of posaconazole was acquired in 10 s. EME coupled with MALDI/MS detection provided the advantages of simplicity, rapidity, and high sensitivity. This method was also successfully applied to the determination of posaconazole in human serum samples. In the future, this developed method will be suitable for the high-throughput screening of posaconazole drug levels in human serum samples.

## Figures and Tables

**Table 1:** Intraday and interday precision and accuracy for EME-MALDI/MS analysis of posaconazole (*n* = 5).

Analyte	Concentration (μM)	Intraday		Interday	
		Precision ^a^ (%)	Accuracy ^b^ (%)	Precision ^a^ (%)	Accuracy ^b^ (%)
Posaconazole	0.5	11.3	4.7	11.6	3.9
	5	10.9	9.1	12.5	8.9
	15	9.3	−4.0	7.4	1.4

^a^ Precision expressed as RSD. ^b^ Accuracy expressed as relative error.

## Data Availability

Not applicable here.
